# Methyl 3,5-bis(cyclo­hexyl­meth­oxy)benzoate

**DOI:** 10.1107/S1600536814004607

**Published:** 2014-03-08

**Authors:** Peter W. R. Corfield, Michele L. Paccagnini, Amy M. Balija

**Affiliations:** a Department of Chemistry, Fordham University, 441 East Fordham Road, Bronx, NY 10458, USA

## Abstract

In the title compound, C_22_H_32_O_4_, the atoms of the methyl ester group and the alk­oxy O atoms are all coplanar with the central aromatic ring, with an r.m.s. deviation of 0.008 Å. Bonds to the methyl­ene and cyclo­hexyl groups are also very close to this plane, so that the mol­ecule is essentially flat, apart from the cyclo­hexyl groups. The mean planes through the cyclo­hexyl groups are tilted by 30.08 (9) and 36.14 (7)° with respect to the central aromatic ring. In the crystal, pairs of mol­ecules linked by C—H⋯O hydrogen bonds form planar units which are stacked along the *a* axis, with an average inter­planar distance of 3.549 (2) Å. Stacking appears to be stabilized by further weak C—H⋯O hydrogen bonds.

## Related literature   

The title compound was synthesized as a monomer for novel dendrimers, as part of a continuing study of how dendrimers effectively complex with organic pollutants in aqueous environments. For a project review, see: Monaco *et al.* (2013 ▶); Corfield & Balija (2013 ▶). For a review of the role of C—H⋯O hydrogen bonds in organic reactions, see: Johnston & Cheong (2013 ▶). For an example of an organic crystal structure involving the cyclo­hexyl­meth­oxy­benzene fragment, see: Yang *et al.* (2008 ▶).
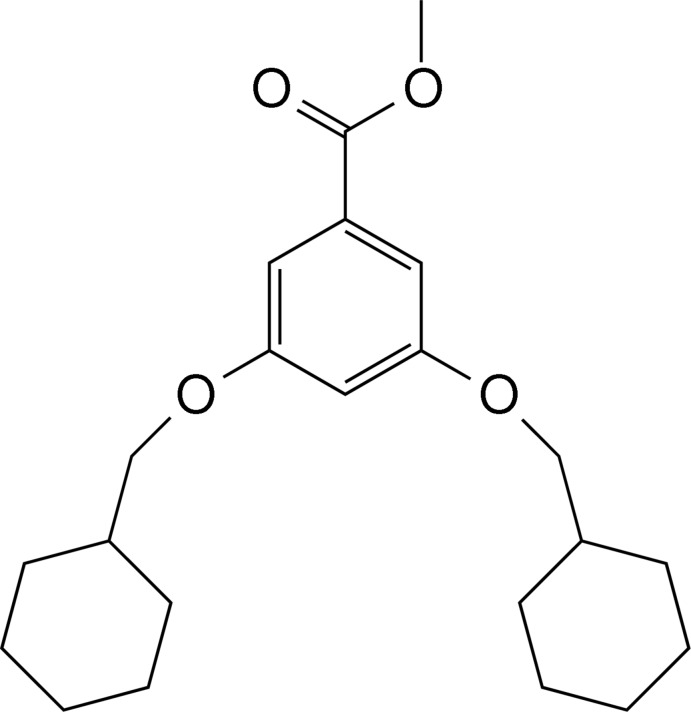


## Experimental   

### 

#### Crystal data  

C_22_H_32_O_4_
*M* *_r_* = 360.48
Triclinic, 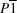

*a* = 6.649 (1) Å
*b* = 12.668 (1) Å
*c* = 12.873 (1) Å
α = 87.64 (1)°
β = 79.46 (1)°
γ = 75.06 (1)°
*V* = 1029.9 (2) Å^3^
*Z* = 2
Mo *K*α radiation
μ = 0.08 mm^−1^
*T* = 298 K
0.75 × 0.75 × 0.53 mm

#### Data collection  

Enraf–Nonius CAD-4 diffractometer
5155 measured reflections
4051 independent reflections
3013 reflections with *I* > 2σ(*I*)
*R*_int_ = 0.008
3 standard reflections every 120 min intensity decay: 1.3 (5)%

#### Refinement  

*R*[*F* ^2^ > 2σ(*F* ^2^)] = 0.044
*wR*(*F* ^2^) = 0.128
*S* = 1.03
4051 reflections
237 parameters
H-atom parameters constrained
Δρ_max_ = 0.17 e Å^−3^
Δρ_min_ = −0.14 e Å^−3^

### 

Data collection: *CAD-4* (Enraf–Nonius, 1994 ▶); cell refinement: *CAD-4*; data reduction: followed procedures in Corfield *et al.* (1973 ▶) and data were averaged with a local version of *SORTAV* (Blessing, 1989 ▶); program(s) used to solve structure: *SHELXS97* (Sheldrick, 2008 ▶); program(s) used to refine structure: *SHELXL97* (Sheldrick, 2008 ▶); molecular graphics: *ORTEPIII* (Burnett & Johnson, 1996 ▶); software used to prepare material for publication: *SHELXL97*.

## Supplementary Material

Crystal structure: contains datablock(s) I. DOI: 10.1107/S1600536814004607/pk2519sup1.cif

Structure factors: contains datablock(s) I. DOI: 10.1107/S1600536814004607/pk2519Isup2.hkl

Supporting information file. DOI: 10.1107/S1600536814004607/pk2519Isup3.cml

CCDC reference: 989172

Additional supporting information:  crystallographic information; 3D view; checkCIF report

## Figures and Tables

**Table 1:** Hydrogen-bond geometry (Å, °)

*D*—H⋯*A*	*D*—H	H⋯*A*	*D*⋯*A*	*D*—H⋯*A*
C8—H8*B*⋯O4^i^	0.96	2.58	3.409 (2)	145
C16—H16*A*⋯O3^ii^	0.97	2.71	3.573 (2)	148
C18—H18*A*⋯O3^ii^	0.97	2.72	3.590 (2)	149

Symmetry codes: (i) 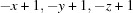; (ii) 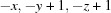.

**Fig. 1.**
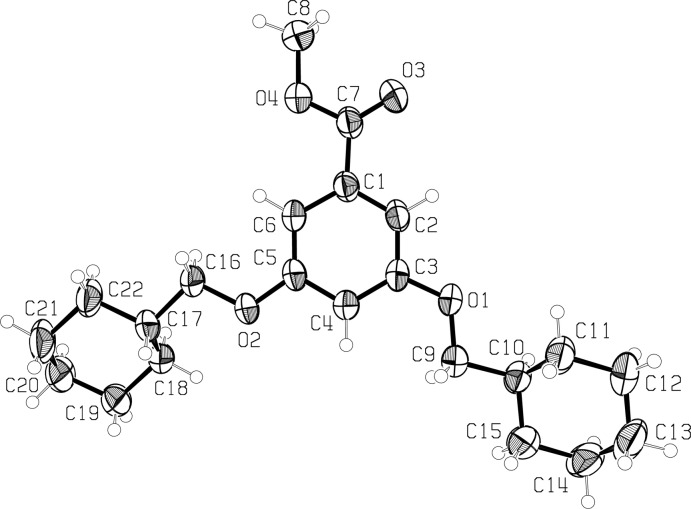
The molecular structure of the title molecule, with ellipsoids at the 50% level.

**Fig. 2.**
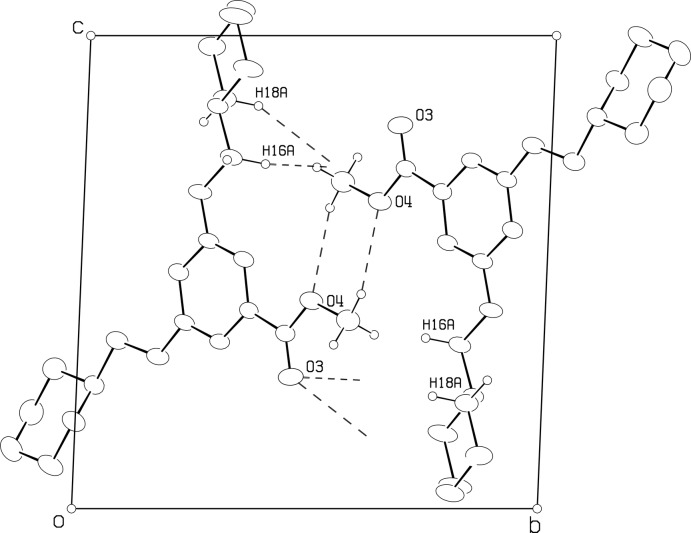
Packing of the title complex, viewed along the a* axis, with ellipsoid outlines at 30% probability. Proposed hydrogen bonds are shown as dashed lines. Hydrogen bonds from O3, C16A and C18A are to molecules translated by -a.

**Fig. 3.**
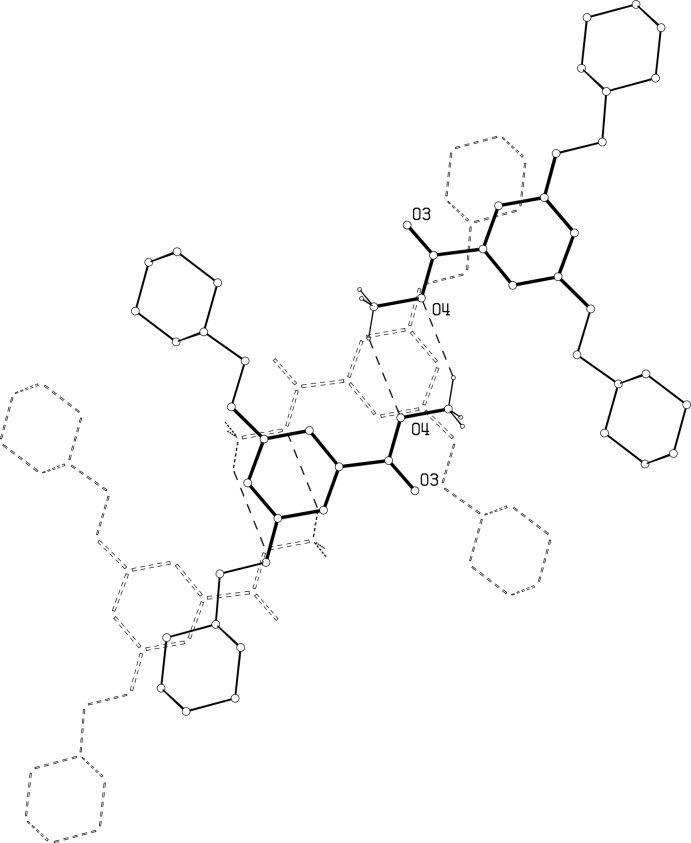
View of the hydrogen-bonded pair of molecules perpendicular to the central molecular plane. The dashed molecules represent a molecular pair unit translated by -a.

**Fig. 4.**
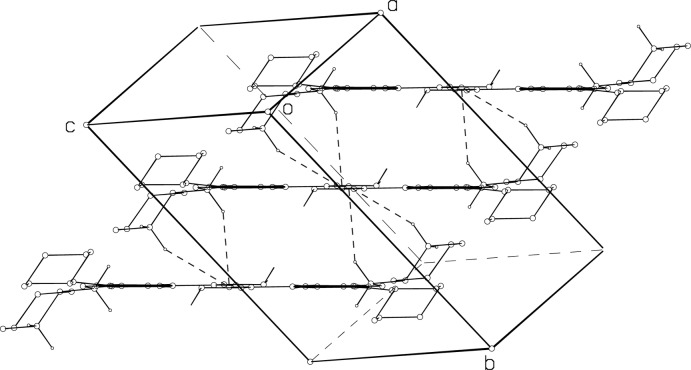
Stacking of molecular pairs related by translations along the a axis. The dashed lines represent the proposed long C—H···O hydrogen bonds. This figure is related to Fig. 3 by rotation of 90° about the horizontal axis.

## supplementary crystallographic information

### 1.1. Synthesis and crystallization

The reaction was performed under an argon gas atmosphere with oven dried glassware. Reagents were obtained from Aldrich and used without further purification. Eluent solvent ratios are reported in v/v.

^1^H NMR spectra were recorded at 300 MHz and ^13^C NMR spectra were recorded at 75 MHz on a Bruker AV-300 High Performance Digital NMR Spectrometer. Chemical shifts are reported in parts per million (ppm) and coupling constants in Hertz (Hz). ^1^H NMR spectra obtained in CDCl_3_ were referenced to 7.26 ppm and ^13^C NMR spectra obtained in CDCl_3_ were referenced to 77.2 ppm. Mass spectra were obtained from University of Illinois Mass Spectrometry Center (Micromass Q-TOF Ultra, ESI).

To a heterogeneous mixture of 8.50 g (61.5 mmol) of K_2_CO_3_ in DMF (37.5 mL) were added 5.00 g (29.7 mmol) of methyl 3,5-di­hydroxy benzoate. After 2 hours, 8.80 mL (63.1 mmol) of bromo­methyl­cyclo­hexane were added over 10 min and the reaction heated at 80^o^C for 3 h. Upon cooling the reaction to room temperature, ethyl acetate (100 mL) was added and the organic layer was washed with water (5X, 70 mL) and brine (1X, 70 mL). The organic layer was dried with anhydrous sodium sulfate and the solvent was removed in vacuo. The resulting mixture of methyl 3-cyclo­hexyl­meth­oxy-5-hy­droxy­benzoate and methyl 3,5-bis­(cyclo­hexyl­meth­oxy)­benzoate was separated by column chromatography (silica gel, petroleum ether:di­ethyl ether, 1:1). The title product was obtained as a yellow oil and allowed to sit undisturbed over several months, when colorless crystals separated, mp 70.6-72.8°C.

^1^H NMR peaks δ: 7.15 (d, *J* = 2.3, 2H), 6.63 (t, *J* = 2.3, 1H), 3.91 (s, 3H), 3.77 (d, *J* = 6.2, 4H), 1.88-1.03 (m, 22H). ^13^C NMR peaks δ: 167.00, 160.6, 132.0, 107.9, 107.0, 73.8, 52.3, 37.6, 29.8, 26.5, 25.8. HRMS-ESI: m/z [M + H]^+^ C_22_H_33_O_4_ 361.2390; found 361.2379.

### 1.2. Refinement

Both forms of the 0 1 0 and of the 0 0 1 reflections were partially obscured by the beam stop, and were omitted from the refinements. H atoms were constrained to idealized positions with C—H distances of 0.93Å for the aromatic H atoms, 0.96Å for the methyl H atoms, 0.97Å for the secondary H atoms and 0.98Å for the tertiary H atoms on C10 and C17. The orientation of the methyl group was determined by calculation of electron density in the toroid that should contain the H atoms of the idealized methyl group. The U_eq_ values for all H atoms were fixed at 1.2 times the U_iso_ of their bonded C atoms.

### 2. Comment

Dendrimers are macromolecules prepared in a stepwise fashion from monomer units and a core molecule. This work is part of a larger study examining how the the modification of functional groups in the monomer impacts the physical and chemical properties of the resulting dendrimer. The title compound is an inter­mediate for a novel cyclo­hexane based dendrimer (Monaco *et al.*, 2013; Corfield and Balija, 2013).

In the title compound, C_22_H_32_O_4_, the four atoms of the methyl ester group and the two oxygen atoms of the 3,5 alk­oxy substituents are all coplanar with the central aromatic ring, with a dihedral angle of the ester group to the ring of only 0.7 (1)°. Bonds to the cyclo­hexyl groups are also close to this plane, with torsional angles C2—C3—O1—C9 and C3—O1—C9—C10 of 172.88 (15)° and 179.66 (14)° respectively, and C6—C5—O2—C16 and C5—O2—C16—C17 angles of 3.4 (3)° and 175.59 (14)° respectively. The C10—C15 and C17—C22 cyclo­hexyl groups are oriented respectively away from and towards the methyl ester group on C1 (Fig. 1), and their mean planes are tilted 30.08 (9)° and 36.14 (7)° to the central aromatic ring. A similar extended conformation for the cyclo­hexyl­meth­oxy substituent in a related compound is found in Yang *et al.* (2008).

Steric repulsion between methyl­ene hydrogen atoms of the alk­oxy groups and ring protons leads to opening of the exterior ring angles to 124.6 (1)° and 24.8 (1)°, and of the bond angles at the ether oxygen atoms to 118.7 (1)° and 117.9 (1)°.

Pairs of molecules are connected by weak C—H··· O hydrogen bonds across the center of symmetry at (1 - *x*, 1 - *y*, 1 - *z*). (Figs. 2 and 3) The central planes of the symmetry-related molecules are almost coplanar, with a perpendicular distance between them of 0.105 (3)Å. The molecular pairs are stacked along the *a* axis, with average inter­planar spacing of 3.549 (2) Å. (Fig. 4) There are no obvious π—π inter­actions to explain the short stacking distance. We propose that part of the inter­planar inter­action arises from the presence of long C—H···O hydrogen bonds between O3 and methyl­ene and cyclo­hexyl hydrogen atoms H16A and H18A. (See Table 1) Such non-classical hydrogen bonds are frequently invoked in recent publications in this journal, and their impact on reaction stereochemistry is reviewed in Johnston and Cheong (2013).

### Crystal data

**Table:**

C_22_H_32_O_4_	*Z* = 2
*M**_r_* = 360.48	*F*(000) = 392
Triclinic, *P*1	*D*_x_ = 1.162 Mg m^−^^3^
Hall symbol: -P 1	Melting point: 344.8 K
*a* = 6.649 (1) Å	Mo *K*α radiation, λ = 0.71070 Å
*b* = 12.668 (1) Å	Cell parameters from 25 reflections
*c* = 12.873 (1) Å	θ = 3.2–9.7°
α = 87.64 (1)°	µ = 0.08 mm^−^^1^
β = 79.46 (1)°	*T* = 298 K
γ = 75.06 (1)°	Block, colourless
*V* = 1029.9 (2) Å^3^	0.75 × 0.75 × 0.53 mm

### Data collection

**Table:**

Enraf–Nonius CAD-4 diffractometer	*R*_int_ = 0.008
Radiation source: fine-focus sealed tube	θ_max_ = 26.0°, θ_min_ = 2.3°
Graphite monochromator	*h* = −1→8
θ/2θ scans	*k* = −15→15
5155 measured reflections	*l* = −15→15
4051 independent reflections	3 standard reflections every 120 min
3013 reflections with *I* > 2σ(*I*)	intensity decay: 1.3(5)

### Refinement

**Table:**

Refinement on *F*^2^	Secondary atom site location: difference Fourier map
Least-squares matrix: full	Hydrogen site location: inferred from neighbouring sites
*R*[*F*^2^ > 2σ(*F*^2^)] = 0.044	H-atom parameters constrained
*wR*(*F*^2^) = 0.128	*w* = 1/[σ^2^(*F*_o_^2^) + (0.033*P*)^2^ + 0.270*P*] where *P* = (*F*_o_^2^ + 2*F*_c_^2^)/3
*S* = 1.03	(Δ/σ)_max_ = 0.002
4051 reflections	Δρ_max_ = 0.17 e Å^−^^3^
237 parameters	Δρ_min_ = −0.14 e Å^−^^3^
0 restraints	Extinction correction: *SHELXL97* (Sheldrick, 2008), Fc^*^=kFc[1+0.001xFc^2^λ^3^/sin(2θ)]^-1/4^
Primary atom site location: structure-invariant direct methods	Extinction coefficient: 0.011 (2)

### Special details

**Table:**

Geometry. All e.s.d.'s (except the e.s.d. in the dihedral angle between two l.s. planes) are estimated using the full covariance matrix. The cell e.s.d.'s are taken into account individually in the estimation of e.s.d.'s in distances, angles and torsion angles; correlations between e.s.d.'s in cell parameters are only used when they are defined by crystal symmetry. An approximate (isotropic) treatment of cell e.s.d.'s is used for estimating e.s.d.'s involving l.s. planes.
Refinement. Refinement of *F*^2^ against ALL reflections. The weighted *R*-factor *wR* and goodness of fit *S* are based on *F*^2^, conventional *R*-factors *R* are based on *F*, with *F* set to zero for negative *F*^2^. The threshold expression of *F*^2^ > σ(*F*^2^) is used only for calculating *R*-factors(gt) *etc*. and is not relevant to the choice of reflections for refinement. *R*-factors based on *F*^2^ are statistically about twice as large as those based on *F*, and *R*-factors based on ALL data will be even larger.

### Fractional atomic coordinates and isotropic or equivalent isotropic displacement parameters (Å^2^)

**Table:**

	*x*	*y*	*z*	*U*_iso_*/*U*_eq_	
O1	−0.2343 (2)	0.20265 (10)	0.34241 (8)	0.0613 (3)	
O2	−0.0616 (2)	0.25640 (11)	0.67501 (8)	0.0628 (4)	
O3	0.2878 (2)	0.42136 (12)	0.25302 (9)	0.0756 (4)	
O4	0.37753 (19)	0.44700 (10)	0.40594 (8)	0.0602 (3)	
C1	0.1250 (2)	0.34685 (12)	0.40804 (11)	0.0450 (4)	
C2	0.0091 (3)	0.30219 (13)	0.35244 (11)	0.0486 (4)	
H2	0.0217	0.3118	0.2797	0.058*	
C3	−0.1264 (3)	0.24282 (13)	0.40479 (11)	0.0490 (4)	
C4	−0.1455 (3)	0.22817 (14)	0.51302 (12)	0.0512 (4)	
H4	−0.2355	0.1878	0.5482	0.061*	
C5	−0.0280 (3)	0.27471 (13)	0.56870 (11)	0.0495 (4)	
C6	0.1076 (3)	0.33363 (13)	0.51749 (11)	0.0485 (4)	
H6	0.1862	0.3641	0.5549	0.058*	
C7	0.2697 (3)	0.40810 (13)	0.34645 (12)	0.0483 (4)	
C8	0.5185 (3)	0.50921 (17)	0.35207 (14)	0.0655 (5)	
H8A	0.6079	0.4683	0.2922	0.079*	
H8B	0.6042	0.5236	0.3995	0.079*	
H8C	0.4372	0.5771	0.3286	0.079*	
C9	−0.3584 (3)	0.13030 (15)	0.38765 (12)	0.0554 (4)	
H9A	−0.4655	0.1661	0.4459	0.067*	
H9B	−0.2692	0.0658	0.4145	0.067*	
C10	−0.4621 (3)	0.09842 (14)	0.30275 (12)	0.0516 (4)	
H10	−0.5436	0.1657	0.2746	0.062*	
C11	−0.3030 (3)	0.03691 (16)	0.21158 (14)	0.0621 (5)	
H11A	−0.2114	0.0823	0.1795	0.075*	
H11B	−0.2157	−0.0286	0.2379	0.075*	
C12	−0.4151 (4)	0.00567 (19)	0.12867 (16)	0.0777 (6)	
H12A	−0.3110	−0.0377	0.0735	0.093*	
H12B	−0.4894	0.0714	0.0967	0.093*	
C13	−0.5711 (4)	−0.05892 (19)	0.17717 (19)	0.0862 (7)	
H13A	−0.6463	−0.0733	0.1237	0.103*	
H13B	−0.4946	−0.1286	0.2016	0.103*	
C14	−0.7281 (4)	0.00134 (19)	0.26845 (18)	0.0795 (6)	
H14A	−0.8190	−0.0444	0.3005	0.095*	
H14B	−0.8163	0.0670	0.2428	0.095*	
C15	−0.6158 (3)	0.03203 (17)	0.35076 (15)	0.0665 (5)	
H15A	−0.7197	0.0742	0.4067	0.080*	
H15B	−0.5395	−0.0339	0.3816	0.080*	
C16	0.0433 (3)	0.30659 (15)	0.73897 (11)	0.0525 (4)	
H16A	0.0098	0.3849	0.7276	0.063*	
H16B	0.1953	0.2778	0.7200	0.063*	
C17	−0.0299 (3)	0.28262 (14)	0.85363 (11)	0.0500 (4)	
H17	0.0038	0.2032	0.8626	0.060*	
C18	−0.2652 (3)	0.32768 (16)	0.88989 (12)	0.0571 (4)	
H18A	−0.3025	0.4059	0.8784	0.069*	
H18B	−0.3411	0.2949	0.8482	0.069*	
C19	−0.3324 (3)	0.3047 (2)	1.00661 (13)	0.0713 (6)	
H19A	−0.4822	0.3385	1.0281	0.086*	
H19B	−0.3101	0.2265	1.0167	0.086*	
C20	−0.2097 (3)	0.3479 (2)	1.07503 (13)	0.0755 (6)	
H20A	−0.2480	0.4271	1.0729	0.091*	
H20B	−0.2473	0.3259	1.1476	0.091*	
C21	0.0244 (3)	0.3063 (2)	1.03912 (14)	0.0855 (7)	
H21A	0.0658	0.2281	1.0511	0.103*	
H21B	0.0976	0.3409	1.0807	0.103*	
C22	0.0908 (3)	0.3291 (2)	0.92233 (13)	0.0706 (6)	
H22A	0.0649	0.4073	0.9117	0.085*	
H22B	0.2413	0.2969	0.9010	0.085*	

### Atomic displacement parameters (Å^2^)

**Table:**

	*U*^11^	*U*^22^	*U*^33^	*U*^12^	*U*^13^	*U*^23^
O1	0.0823 (8)	0.0788 (8)	0.0403 (6)	−0.0437 (7)	−0.0227 (6)	0.0013 (5)
O2	0.0878 (9)	0.0868 (9)	0.0294 (5)	−0.0490 (7)	−0.0117 (5)	−0.0017 (5)
O3	0.1073 (11)	0.0992 (10)	0.0400 (7)	−0.0588 (9)	−0.0200 (6)	0.0126 (6)
O4	0.0700 (8)	0.0812 (8)	0.0408 (6)	−0.0389 (7)	−0.0108 (5)	−0.0017 (5)
C1	0.0534 (9)	0.0468 (8)	0.0358 (7)	−0.0132 (7)	−0.0093 (6)	−0.0030 (6)
C2	0.0623 (10)	0.0546 (9)	0.0326 (7)	−0.0175 (8)	−0.0140 (7)	−0.0004 (6)
C3	0.0596 (10)	0.0563 (9)	0.0364 (7)	−0.0190 (8)	−0.0150 (7)	−0.0059 (7)
C4	0.0612 (10)	0.0616 (10)	0.0379 (8)	−0.0272 (8)	−0.0092 (7)	−0.0034 (7)
C5	0.0618 (10)	0.0586 (10)	0.0311 (7)	−0.0193 (8)	−0.0094 (6)	−0.0058 (6)
C6	0.0580 (9)	0.0581 (10)	0.0349 (7)	−0.0208 (8)	−0.0122 (7)	−0.0056 (6)
C7	0.0581 (9)	0.0508 (9)	0.0376 (8)	−0.0144 (7)	−0.0108 (7)	−0.0023 (6)
C8	0.0700 (12)	0.0821 (13)	0.0543 (10)	−0.0387 (10)	−0.0084 (9)	−0.0001 (9)
C9	0.0628 (10)	0.0688 (11)	0.0424 (8)	−0.0274 (9)	−0.0130 (7)	−0.0026 (8)
C10	0.0554 (9)	0.0567 (10)	0.0491 (9)	−0.0192 (8)	−0.0186 (7)	−0.0024 (7)
C11	0.0644 (11)	0.0728 (12)	0.0551 (10)	−0.0219 (9)	−0.0170 (8)	−0.0115 (8)
C12	0.0951 (15)	0.0896 (15)	0.0598 (11)	−0.0326 (12)	−0.0261 (11)	−0.0183 (10)
C13	0.1146 (18)	0.0749 (14)	0.0933 (16)	−0.0410 (13)	−0.0549 (14)	−0.0056 (12)
C14	0.0806 (14)	0.0876 (15)	0.0923 (16)	−0.0464 (12)	−0.0389 (12)	0.0143 (12)
C15	0.0657 (12)	0.0796 (13)	0.0647 (11)	−0.0324 (10)	−0.0199 (9)	0.0060 (10)
C16	0.0600 (10)	0.0700 (11)	0.0344 (8)	−0.0261 (8)	−0.0110 (7)	−0.0060 (7)
C17	0.0632 (10)	0.0591 (10)	0.0320 (7)	−0.0201 (8)	−0.0122 (7)	−0.0030 (6)
C18	0.0603 (10)	0.0788 (12)	0.0414 (8)	−0.0310 (9)	−0.0132 (7)	0.0013 (8)
C19	0.0726 (12)	0.1068 (16)	0.0428 (9)	−0.0418 (12)	−0.0042 (8)	0.0003 (9)
C20	0.0836 (14)	0.1131 (17)	0.0355 (9)	−0.0389 (13)	−0.0022 (8)	−0.0136 (9)
C21	0.0782 (14)	0.148 (2)	0.0381 (9)	−0.0345 (14)	−0.0178 (9)	−0.0129 (11)
C22	0.0581 (11)	0.1212 (17)	0.0398 (9)	−0.0323 (11)	−0.0101 (8)	−0.0153 (10)

### Geometric parameters (Å, º)

**Table:**

O1—C3	1.3608 (17)	C12—H12A	0.9700
O1—C9	1.4245 (19)	C12—H12B	0.9700
O2—C5	1.3658 (18)	C13—C14	1.506 (3)
O2—C16	1.4322 (17)	C13—H13A	0.9700
O3—C7	1.1959 (18)	C13—H13B	0.9700
O4—C7	1.3256 (18)	C14—C15	1.518 (2)
O4—C8	1.4415 (19)	C14—H14A	0.9700
C1—C2	1.373 (2)	C14—H14B	0.9700
C1—C6	1.399 (2)	C15—H15A	0.9700
C1—C7	1.487 (2)	C15—H15B	0.9700
C2—C3	1.385 (2)	C16—C17	1.511 (2)
C2—H2	0.9300	C16—H16A	0.9700
C3—C4	1.385 (2)	C16—H16B	0.9700
C4—C5	1.396 (2)	C17—C22	1.523 (2)
C4—H4	0.9300	C17—C18	1.512 (2)
C5—C6	1.376 (2)	C17—H17	0.9800
C6—H6	0.9300	C18—C19	1.526 (2)
C8—H8A	0.9600	C18—H18A	0.9700
C8—H8B	0.9600	C18—H18B	0.9700
C8—H8C	0.9600	C19—C20	1.508 (2)
C9—C10	1.513 (2)	C19—H19A	0.9700
C9—H9A	0.9700	C19—H19B	0.9700
C9—H9B	0.9700	C20—C21	1.499 (3)
C10—C15	1.518 (2)	C20—H20A	0.9700
C10—C11	1.516 (2)	C20—H20B	0.9700
C10—H10	0.9800	C21—C22	1.525 (2)
C11—C12	1.526 (2)	C21—H21A	0.9700
C11—H11A	0.9700	C21—H21B	0.9700
C11—H11B	0.9700	C22—H22A	0.9700
C12—C13	1.514 (3)	C22—H22B	0.9700
			
C3—O1—C9	118.71 (12)	C14—C13—H13B	109.3
C5—O2—C16	117.86 (12)	C12—C13—H13B	109.3
C7—O4—C8	116.24 (12)	H13A—C13—H13B	107.9
C2—C1—C6	120.88 (14)	C13—C14—C15	110.87 (17)
C2—C1—C7	116.97 (13)	C13—C14—H14A	109.5
C6—C1—C7	122.15 (13)	C15—C14—H14A	109.5
C1—C2—C3	119.89 (14)	C13—C14—H14B	109.5
C1—C2—H2	120.1	C15—C14—H14B	109.5
C3—C2—H2	120.1	H14A—C14—H14B	108.1
O1—C3—C4	124.58 (14)	C10—C15—C14	111.39 (16)
O1—C3—C2	115.13 (13)	C10—C15—H15A	109.3
C4—C3—C2	120.28 (14)	C14—C15—H15A	109.3
C3—C4—C5	119.23 (15)	C10—C15—H15B	109.3
C3—C4—H4	120.4	C14—C15—H15B	109.3
C5—C4—H4	120.4	H15A—C15—H15B	108.0
O2—C5—C6	124.79 (13)	O2—C16—C17	108.62 (13)
O2—C5—C4	114.21 (14)	O2—C16—H16A	110.0
C6—C5—C4	121.00 (13)	C17—C16—H16A	110.0
C5—C6—C1	118.70 (14)	O2—C16—H16B	110.0
C5—C6—H6	120.6	C17—C16—H16B	110.0
C1—C6—H6	120.6	H16A—C16—H16B	108.3
O3—C7—O4	123.11 (15)	C16—C17—C22	109.33 (14)
O3—C7—C1	123.90 (14)	C16—C17—C18	113.02 (14)
O4—C7—C1	112.99 (12)	C22—C17—C18	109.76 (13)
O4—C8—H8A	109.5	C16—C17—H17	108.2
O4—C8—H8B	109.5	C22—C17—H17	108.2
H8A—C8—H8B	109.5	C18—C17—H17	108.2
O4—C8—H8C	109.5	C19—C18—C17	111.55 (15)
H8A—C8—H8C	109.5	C19—C18—H18A	109.3
H8B—C8—H8C	109.5	C17—C18—H18A	109.3
O1—C9—C10	108.22 (13)	C19—C18—H18B	109.3
O1—C9—H9A	110.1	C17—C18—H18B	109.3
C10—C9—H9A	110.1	H18A—C18—H18B	108.0
O1—C9—H9B	110.1	C20—C19—C18	111.71 (15)
C10—C9—H9B	110.1	C20—C19—H19A	109.3
H9A—C9—H9B	108.4	C18—C19—H19A	109.3
C9—C10—C15	109.83 (14)	C20—C19—H19B	109.3
C9—C10—C11	112.77 (14)	C18—C19—H19B	109.3
C15—C10—C11	110.74 (15)	H19A—C19—H19B	107.9
C9—C10—H10	107.8	C21—C20—C19	111.69 (16)
C15—C10—H10	107.8	C21—C20—H20A	109.3
C11—C10—H10	107.8	C19—C20—H20A	109.3
C12—C11—C10	110.79 (15)	C21—C20—H20B	109.3
C12—C11—H11A	109.5	C19—C20—H20B	109.3
C10—C11—H11A	109.5	H20A—C20—H20B	107.9
C12—C11—H11B	109.5	C20—C21—C22	111.75 (17)
C10—C11—H11B	109.5	C20—C21—H21A	109.3
H11A—C11—H11B	108.1	C22—C21—H21A	109.3
C11—C12—C13	111.22 (17)	C20—C21—H21B	109.3
C11—C12—H12A	109.4	C22—C21—H21B	109.3
C13—C12—H12A	109.4	H21A—C21—H21B	107.9
C11—C12—H12B	109.4	C17—C22—C21	111.58 (16)
C13—C12—H12B	109.4	C17—C22—H22A	109.3
H12A—C12—H12B	108.0	C21—C22—H22A	109.3
C14—C13—C12	111.78 (17)	C17—C22—H22B	109.3
C14—C13—H13A	109.3	C21—C22—H22B	109.3
C12—C13—H13A	109.3	H22A—C22—H22B	108.0
			
C2—C3—O1—C9	172.88 (15)	C4—C5—O2—C16	−176.46 (15)
C4—C3—O1—C9	−7.4 (3)	C6—C5—O2—C16	3.3 (3)
C3—O1—C9—C10	179.66 (14)	C5—O2—C16—C17	175.59 (14)
O1—C9—C10—C11	62.55 (19)	O2—C16—C17—C18	−61.10 (19)
O1—C9—C10—C15	−173.41 (15)	O2—C16—C17—C22	176.33 (15)

### Hydrogen-bond geometry (Å, º)

**Table:**

*D*—H···*A*	*D*—H	H···*A*	*D*···*A*	*D*—H···*A*
C8—H8*B*···O4^i^	0.96	2.58	3.409 (2)	145
C16—H16*A*···O3^ii^	0.97	2.71	3.573 (2)	148
C18—H18*A*···O3^ii^	0.97	2.72	3.590 (2)	149

Symmetry codes: (i) −*x*+1, −*y*+1, −*z*+1; (ii) −*x*, −*y*+1, −*z*+1.
